# Lipid metabolism has been good to me

**DOI:** 10.1016/j.jbc.2021.100786

**Published:** 2021-05-18

**Authors:** George M. Carman

**Affiliations:** Department of Food Science and the Rutgers Center for Lipid Research, New Jersey Institute for Food, Nutrition, and Health, Rutgers University, New Brunswick, New Jersey, USA

**Keywords:** lipid, metabolism, phospholipid, phosphatidate, diacylglycerol, triacylglycerol, phosphatase, AE, associate editor, ASBMB, American Society for Biochemistry and Molecular Biology, BBA, *Biochimica et Biophysica Acta*, DAG, diacylglycerol, EE, executive editor, EIC, editor-in-chief, FASEB, Federation of American Societies for Experimental Biology, JBC, *Journal of Biological Chemistry*, JLR, *Journal of Lipid Research*, PA, phosphatidic acid, PC, physiological chemistry, PI, phosphatidylinositol, PS, phosphatidylserine, RCLR, Rutgers Center for Lipid Research

## Abstract

My career in research has flourished through hard work, supportive mentors, and outstanding mentees and collaborators. The Carman laboratory has contributed to the understanding of lipid metabolism through the isolation and characterization of key lipid biosynthetic enzymes as well as through the identification of the enzyme-encoding genes. Our findings from yeast have proven to be invaluable to understand regulatory mechanisms of human lipid metabolism. Several rewarding aspects of my career have been my service to the *Journal of Biological Chemistry* as an editorial board member and Associate Editor, the National Institutes of Health as a member of study sections, and national and international scientific meetings as an organizer. I advise early career scientists to not assume anything, acknowledge others’ accomplishments, and pay it forward.

Reflections articles, which were introduced to commemorate the *Journal of Biological Chemistry* (JBC) Centenary in 2005, highlight the careers of scientists who have made the journal a home for their work. Until now, I have authored 105 papers in the JBC. I am truly honored that Herbert Tabor, a long-time editor-in-chief (EIC) of the journal, invited me to write my story.

## Education and how I wound up at Rutgers University

I grew up in humble surroundings in Jersey City, New Jersey. My mother was the only one in the family who finished high school prior to my older sister, and I was the first in my family to attend college. I always did well in biology and chemistry classes, and so, I gravitated to those subjects. I went to William Paterson College (BA, 1972) where I was recruited by a swimming coach Arthur Raidy; I swam breast stroke and the individual medley for the college team. I majored in biology and minored in chemistry. John Rosengren (biology teacher) and Ashot Merijanian (chemistry teacher) were particularly encouraging; I wanted to be a college professor like them, and so, I sought an advanced degree.

I pursued an MS degree (1974) in biology at Seton Hall University where I served as a teaching assistant in biology and conducted a research project on the expression of mannitol dehydrogenase activity in *Agrobacterium*. A member of my thesis committee, Elvira Doman (now a retired program director of the National Science Foundation), encouraged me to pursue a research career in biochemistry; she continues to be a mentor and friend. My MS degree advisor, John Keller, introduced me to laboratory research and gave me an appreciation for science in a broader context by encouraging me to attend meetings of the Theobald Smith Society for Microbiology (local branch of the American Society for Microbiology). He also encouraged me to apply to the University of Massachusetts (PhD, 1977) where Robert Levin had an open graduate research position in food microbiology. I did not realize that my PhD degree would be in Food Science or that I would have to take undergraduate and graduate courses in the subject to pass the qualifying examination. It was during my doctoral research that I first learned how to purify and characterize an enzyme. I worked on a pyridoxal-5-phosphate-dependent enzyme named tyrosine phenol-lyase, an enzyme from the bacterium *Aeromonas phenologenes* that produces the toxin phenol in fish (1). There are three products of this reaction, namely phenol (identified by gas chromatography), pyruvate (identified by 2, 4-dinitrophenylhydrazine derivatization), and ammonia (identified by nesslerization); Levin had me confirm the stoichiometric formation of each of the products. A routine assay measured the formation of pyruvate from tyrosine by following a decrease in A_340 nm_ in the presence of lactate dehydrogenase and NADH (1). Under Levin’s tutelage, I had to improvise for basic biochemical equipment; for example, we made chromatography columns out of glass pipes and used makeshift PAGE units and casting tubes. He also made the laboratory recycle all solvents. This training in frugality was very helpful when I started my own laboratory without enough funds for supplies and equipment.

Levin had done a sabbatical at Baylor College of Medicine and glorified Houston as a hotbed for basic research. So, when it came time to seek a postdoctoral position, I applied to biochemistry laboratories in Houston. One laboratory that I applied to was that of Bill Dowhan (2)† at the University of Texas Medical School. Bill’s laboratory was studying the structure and function of *Escherichia coli* membranes with an emphasis on the genes and enzymes responsible for synthesizing phospholipids. Just a few years earlier, Singer and Nicholson published the famous fluid mosaic model membrane structure (3), and understanding the regulation of membrane phospholipid synthesis was becoming an area of interest that I wanted to be involved in. I received an offer from Bill by phone, accepted it, and headed for Houston during the first week of January 1977. He later told me that the reason he had accepted me into his laboratory was because I had studied a yellow-colored pyridoxal-5-phosphate-dependent enzyme and he had also studied a similar enzyme, D-serine dehydratase (4). Whereas I thought I would be purifying enzymes involved in phospholipid metabolism, I spent half of my time chemically synthesizing the liponucleotide CDP–diacylglycerol (DAG) and its fatty-acyl analogs for substrate-specificity studies of the CDP–DAG–dependent enzyme phosphatidylserine (PS) synthase (5). I learned a great deal about organic synthesis and became an expert in rotary evaporation. The enzyme kinetic experiments were based on a “surface dilution kinetics” model that Ed Dennis (6)† and colleagues devised for interfacial enzymes (7, 8). To perform the kinetic studies, I developed a continuous spectrophotometric assay whereby the release of CMP from CDP–DAG is coupled to the oxidation of NADH in the presence of CMP kinase, pyruvate kinase, and lactate dehydrogenase (9). Of course, I was familiar with coupled spectrophotometric assays using lactate dehydrogenase from my doctoral studies. Previously, a cumbersome radioactive assay was used to measure PS synthase activity by monitoring the incorporation of water-soluble radiolabeled serine into chloroform-soluble radiolabeled PS (10).

For the newly developed spectrophotometric assay, I had to purify the CMP kinase, which was not commercially available. During this process, I learned many tricks from Bill that I did not know from my doctoral research on tyrosine phenol-lyase. Bill had much experience in purifying enzymes (4, 10, 11, 12, 13, 14), and in fact, he purified the first membrane-associated enzyme, the *E. coli* PS decarboxylase (15). My postdoctoral work merged my interests in enzymology and chemistry, which laid a foundation for much of the biochemical research in my career. One important thing that I learned from Bill was how to listen to laboratory members and let them know that their ideas and opinions were important.

My association with Bill also brought me into the Kennedy (16)† lab family (Bill was a postdoctoral fellow in Eugene Kennedy’s laboratory (2)), which facilitated scientific friendships with Kennedy and with other Kennedy disciples including Ed Dennis, Chris Raetz, Carlos Hirschberg (17)†, and Dennis Voelker. Kennedy is my ultimate idol; he was a disciple of Albert Lehninger, a member of the National Academy of Sciences, and discovered almost all of the enzymes known to be involved in phospholipid synthesis (18, 19, 20). My first PhD student, Tony Fischl, became Kennedy’s last postdoctoral mentee. When Kennedy closed his laboratory and emptied the file cabinets of his reprints, Tony made a collection for me that I bound. In reading each and every paper of his research, I’ve gained much insight into Kennedy’s thinking on how complex questions can be answered with simple experiments. I had a cordial relationship with Kennedy and affectionately called him grandpa when meeting him at scientific meetings.

After my first year in Bill’s laboratory, I began to look for a permanent position because I was worried about my ability to find a faculty position. I applied for positions in numerous biochemistry departments but did not receive a positive response. However, I was offered two positions, one at General Mills in Minneapolis and the other in the Department of Food Science at Rutgers University because of my degree in food science. I was first drawn to General Mills because of a much higher salary and the living environment in Minneapolis, but I was being hired to work on Betty Crocker Potatoes Au Gratin, and this project was not enticing. A faculty position was my real goal, but I wanted a position in biochemistry, not one in food science. Bill was surprised to learn that I would be leaving his laboratory after such a short time, but he recommended that I take a position at Rutgers; he reasoned that it was my best option for a faculty position given my doctoral degree in food science. I accepted the offer from Rutgers because I was being hired for my biochemical expertise and could do any research in the broad area of food science. An extenuating circumstance was that my mother had just been diagnosed with pancreatic cancer and I wanted to be near home in New Jersey. So, after 1.5 years, I left Houston in June 1978 to embark on my independent research career.

## The research that shaped my career

**Figure 1:**
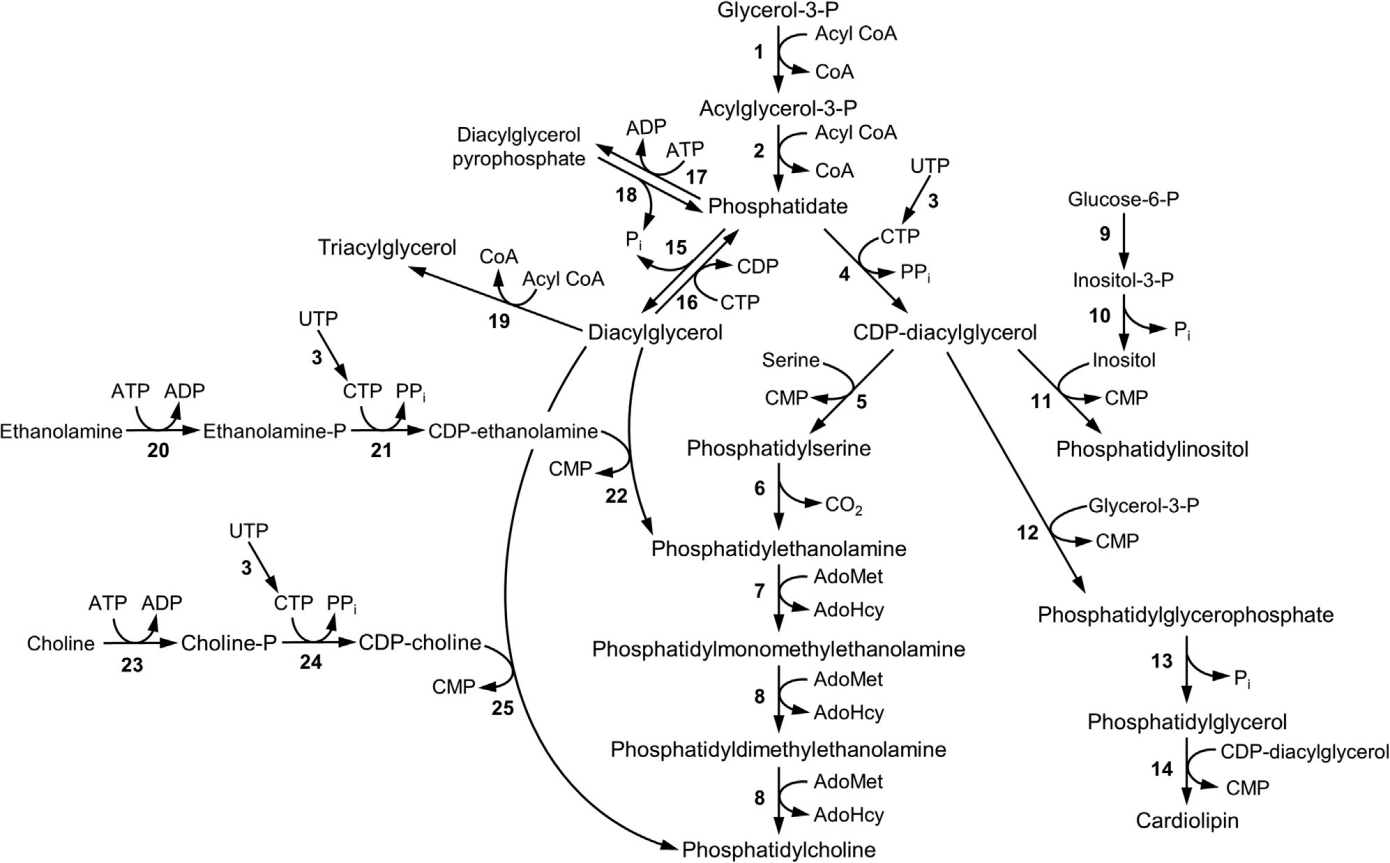
**Phospholipid biosynthetic pathways in *Saccharomyces cerevisiae*.** The indicated reactions are catalyzed by the following enzymes: 1, glycerol-3-P acyltransferase; 2, acylglycerol-3-P acyltransferase; 3, CTP synthetase; 4, CDP–diacylglycerol synthase; 5, phosphatidylserine synthase; 6, phosphatidylserine decarboxylase; 7, phosphatidylethanolamine methyltransferase; 8, phospholipid methyltransferase; 9, inositol-3-P synthase; 10, inositol-3-P phosphatase; 11, phosphatidylinositol synthase; 12, phosphatidylglycerophosphate synthase; 13, phosphatidylglycerophosphate phosphatase; 14, cardiolipin synthase; 15, phosphatidate phosphatase; 16, diacylglycerol kinase; 17, phosphatidate kinase; 18, diacylglycerol pyrophosphate phosphatase; 19, diacylglycerol acyltransferase; 20, ethanolamine kinase; 21, ethanolamine-P cytidylyltransferase, 22, ethanolamine phosphotransferase; 23, choline kinase; 24, choline-P cytidylyltransferase; 25, choline phosphotransferase.

My startup package at Rutgers included $10,000 for equipment, which I used to purchase a Gilford spectrophotometer, $7000 for supplies, and a graduate student assistant. The supply money was tied to an existing United States Department of Agriculture–New Jersey Agricultural Experiment Station project from the professor I replaced, meaning that I would be required to investigate the postharvest physiology of potatoes and tomatoes. I therefore set off to identify membrane-associated phospholipid biosynthetic enzymes in these foods. I focused on phosphatidylinositol (PI) synthase because of my familiarity with PS synthase; both enzymes utilize CDP–DAG as a substrate (21, 22) (Fig. 1). Moreover, the spectrophotometric assay I had developed for CDP–DAG–dependent enzymes (9) could be used to assay PI synthase, and the affinity chromatography resin CDP–DAG–Sepharose (12) developed by Tim Larson in the Dowhan laboratory was available for its purification. However, I immediately found the complications of enzymatic browning in sliced potatoes and messy locules in cut tomatoes when preparing cell-free extracts. Moreover, the enzyme activity was very low in the minuscule amounts of microsomal membranes isolated from these tissues.

Following the suggestion from Theo Solomos, a horticulturist from the University of Maryland, I switched to germinating soybeans as a plant model because of their high rates of lipid synthesis. From the organism, we were able to isolate and characterize several membrane-associated phospholipid biosynthetic enzymes (23, 24, 25, 26, 27, 28, 29). The publications of this work, most of which was performed with undergraduate students, were essential to my early success at Rutgers. Moreover, the department chair, Stephen Chang, rewarded my productivity by purchasing major equipment for my laboratory. Until then, I had to utilize equipment from other departments at the university. Bob Niederman, whose biochemistry laboratory is located on the Busch campus across the Raritan River, was very kind in allowing me to use his scintillation counter at nights and on weekends. We also published some work on phospholipid biosynthetic enzymes from the gram-positive bacterium *Clostridium perfringens* (30, 31, 32, 33). Unfortunately, I was unable to obtain federal funding to study phospholipid metabolism in soybeans or in bacteria.

A turning point in my career came in 1980 when I was introduced to Gary Sanderson, Vice President of Technologies at Universal Foods (producers of Red Star Yeast). He suggested that I study phospholipid synthesis in yeast because it might have commercial implications for enhancing or stabilizing membrane function for baking. The company provided me with an unrestricted grant of $5000 and 12 one-pound blocks of frozen compressed yeast. At this time, very little was known about phospholipid synthesis in yeast except for papers published by Bob Lester *et al.* (34, 35, 36, 37, 38, 39, 40). With a hammer and chisel, I chipped off a chunk of yeast from a 1-pound block, prepared microsomal membranes, and assayed them for PI synthase activity. It was gratifying to know that the enzyme was not only present in those membranes but its activity was 20-fold higher when compared with that found in soybean membranes. Yeast became our main experimental system. They have a cell wall, and so I could justify using them as a plant model. As a first step in the purification of PI synthase, we were able to solubilize yeast membranes to extract the enzyme with a high yield of activity (41). With preliminary data in hand, I submitted a grant application (GM028140, Phospholipid metabolism and membrane function) to the National Institutes of Health (NIH) to purify and characterize the enzyme using yeast as a model eukaryote. It was funded with a first-year direct cost budget of $37,295. Within the next few years, we purified the yeast PI synthase (42), PS synthase (43, 44), and CDP–DAG synthase (45) using a modified version of the CDP–DAG–Sepharose affinity resin. This same grant, which received a merit award in 2010, was funded from 1980 to 2020. Switching to yeast in 1980 was fortuitous because of the emerging advances in yeast molecular genetics; it became facile to isolate genes by complementation of their mutations *via* transformation with plasmids containing genomic DNA fragments (46). For genetic experiments, we switched from the commercial yeast to genetically defined haploid strains. The 1-pound blocks of Red Star Yeast remained in my freezer for several years.

In the spring of 1981, I introduced myself to the renowned yeast geneticist Susan Henry at the annual meeting of the American Society of Microbiology. I sought a collaboration because of her work with the *cho1* mutant defective in PS synthase (47, 48). I had in mind that the mutant could be used to clone the structural gene for the enzyme by complementation and the gene overexpression would facilitate the purification of this enzyme. The first encounter did not go as planned as Susan seemed hesitant; I felt that she considered me a competitor. Bill knew Susan and he made a phone call to assuage her concerns, which paved the way for a fruitful collaboration. Two years later, we coauthored a paper in the *Proceedings of the National Academy of Sciences* that reported the cloning of the *CHO1* gene (Susan’s laboratory) along with the purification of its encoded product, PS synthase (my laboratory) (43). This was the first eukaryotic phospholipid synthesis gene cloned and the second eukaryotic phospholipid synthesis enzyme purified, the first being the yeast PI synthase (42). Susan and I have published numerous papers (43, 49, 50, 51, 52, 53, 54, 55, 56, 57, 58) and reviews (59, 60, 61, 62, 63) on the regulation of phospholipid synthesis in yeast with a focus on the water-soluble phospholipid precursors inositol and choline. Central to this work is the Opi1/Ino2-Ino4 (Henry) regulatory circuit discovered in Susan’s laboratory (60, 64, 65, 66).

During his PhD defense from my laboratory in 1987, Mike Homann, who worked on the regulation of the phosphatidic acid (PA)-branch point enzyme CDP–DAG synthase, suggested that the laboratory study the other branch-point enzyme PA phosphatase (Fig. 1). Although PA phosphatase is primarily used for the synthesis of the neutral lipid triacylglycerol, the control of its activity has a significant impact on the synthesis of membrane phospholipids (67, 68). In 1989, my graduate student Yi-Ping Lin purified PA phosphatase (69), and for the next few years, several mentees worked unsuccessfully to identify a mutant defective in the enzyme or to isolate its structural gene. By 1993, we had given up on trying to isolate the gene by a reverse genetics approach because we could not obtain unambiguous protein sequence information by Edman degradation. In that year, Wen-I Wu, who worked on the biochemical regulation of PA phosphatase (70, 71, 72), left her enzyme sample in the −80 °C freezer. This sample would prove to be fortuitous for our future research.

In 1992, I met Odile Ozier-Kalogeropoulos while attending the International Conference of Yeast Genetics and Molecular Biology in Vienna. Odile presented a poster on the cloning of the *URA7* gene that encodes CTP synthetase. Being aware that the enzyme is essential for all organisms to generate CTP required for the synthesis of all membrane phospholipids (Fig. 1), I initiated a collaboration with her with an idea that manipulating *URA7* expression could modulate the cellular levels of CTP and thereby phospholipid synthesis. On the flight back to New Jersey, I outlined a research plan for an NIH grant application that was funded in 1994 (GM050679, Regulation of phospholipid synthesis), renewed several times (26 years total), and merged in 2020 with NIH grant GM028140 for my current MIRA Award (GM136128, Regulation and role of phosphatidate phosphatase in lipid metabolism). Our studies on CTP synthetase revealed that it is regulated by CTP product inhibition and through its nucleotide- and phosphorylation-dependent tetramerization. This regulation governs the cellular levels of CTP and the pathways by which phospholipids are synthesized (73). Much of what we learned about the phosphorylation of CTP synthetase has been adapted to our studies on the phosphorylation of other proteins (PS synthase, choline kinase, DAG kinase, PA phosphatase, and Opi1 repressor) in yeast lipid metabolism (65, 66).

In the next year (1993), I attended the International Conference on the Bioscience of Lipids in Noordwijkerhout, the Netherlands, where I met Josef Wissing. He sought a collaboration to study a plant PA phosphatase that differed from the enzyme purified by Yi-Ping Lin in 1989. Through studies conducted by Wen-I Wu, we learned that a yeast counterpart of the plant enzyme preferred DAG pyrophosphate to PA as substrate, and thus named it as DAG pyrophosphate phosphatase (74). DAG pyrophosphate was a novel phospholipid identified by Wissing as the product of PA kinase (75, 76), which was later shown to play a role as a signaling lipid in plants (77). We also learned that unlike the Mg^2+^-dependent PA phosphatase involved in the synthesis of triacylglycerol, DAG pyrophosphate phosphatase had no cofactor requirement, had an acidic pH optimum, and functioned *via* a completely different reaction mechanism (74, 78, 79). Subsequently, Dave Toke cloned *DPP1*, which encodes this enzyme, using a reverse genetics approach (80). Within days of publication of *DPP1* cloning, I received a phone call from David Eide, an expert in zinc transport, informing me that *DPP1* was highly regulated by zinc limitation (81). This revelation prompted my laboratory to go in a new direction, studying the transcriptional regulation of phospholipid synthesis genes by zinc (82, 83, 84, 85, 86, 87, 88, 89).

In the summer of 2005, I instructed my lab to discard samples in the −80 °C freezer that were dated more than 10 years old and would not be used anymore. While cleaning up the freezer, Gil-Soo Han found PA phosphatase that Wen-I Wu had purified in 1993 and suggested reanalyzing the old enzyme preparation. Being aware of the yeast genome database and advances in MS, we retried to identify proteins in the enzyme preparation. MS analysis of the enzyme separated by SDS-PAGE showed 23 peptides that unambiguously matched the deduced amino acid sequence of the *SMP2* gene identified by the Oshima laboratory in 1993 (90). Gil-Soo purified the product of *SMP2* through its heterologous overexpression in *E. coli* and confirmed that the gene product is the Mg^2+^-dependent PA phosphatase (67). Since the name *SMP2* was not associated with the molecular function of its protein product, it was renamed *PAH1* for phosphatidic acid phosphohydrolase. (*PAP1* could be a better choice if it was not already assigned for the poly(A) polymerase gene). The discoveries of all PA phosphatase genes in yeast are reviewed in Ref. (91).

**Figure 2:**
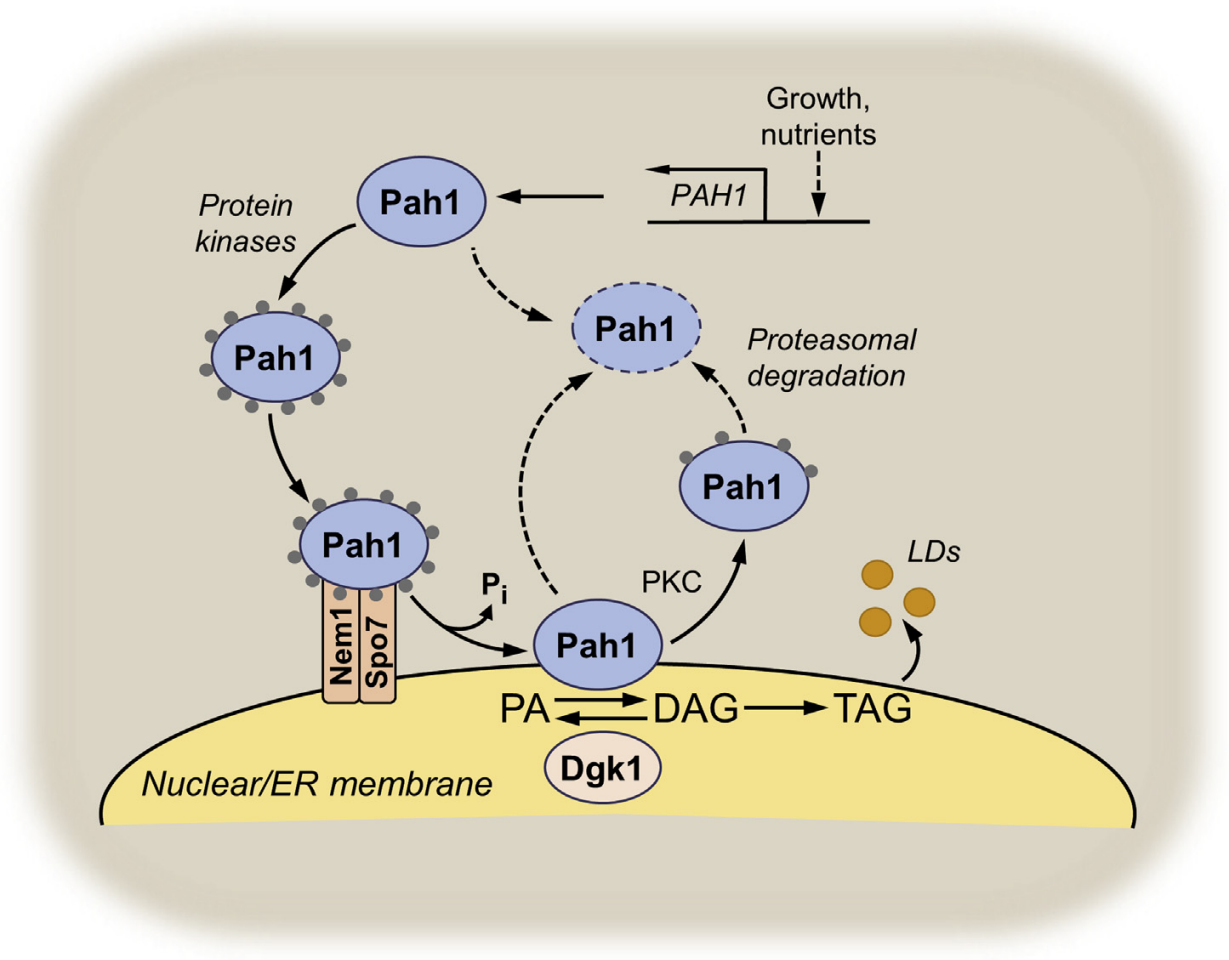
**Model for the regulation of Pah1 PA phosphatase.** The figure depicts a model for the transcriptional and biochemical regulations of Pah1 PA phosphatase in *Saccharomyces cerevisiae*. The expression of the *PAH1* gene is regulated by the growth phase and nutrient status (89, 142). Pah1 is phosphorylated (*small gray circles*) by multiple protein kinases in the cytosol and translocates to the nuclear/ER membrane through its dephosphorylation by the Nem1–Spo7 phosphatase complex (99, 131). Dephosphorylated Pah1 associated with the nuclear/ER membrane *via* its amphipathic helix (131) catalyzes the conversion of PA to DAG (67), which is then acylated to TAG for storage in lipid droplets (LDs). Unphosphorylated/dephosphorylated Pah1 or PKC-phosphorylated Pah1 (143) is degraded by the 20S proteasome (indicated by the *dashed line arrows*) (144). DAG is phosphorylated by the CTP-dependent Dgk1 DAG kinase to produce PA. DAG, diacylglycerol; ER, endoplasmic reticulum; PA, phosphatidic acid; TAG, triacylglycerol.

**Figure 3:**
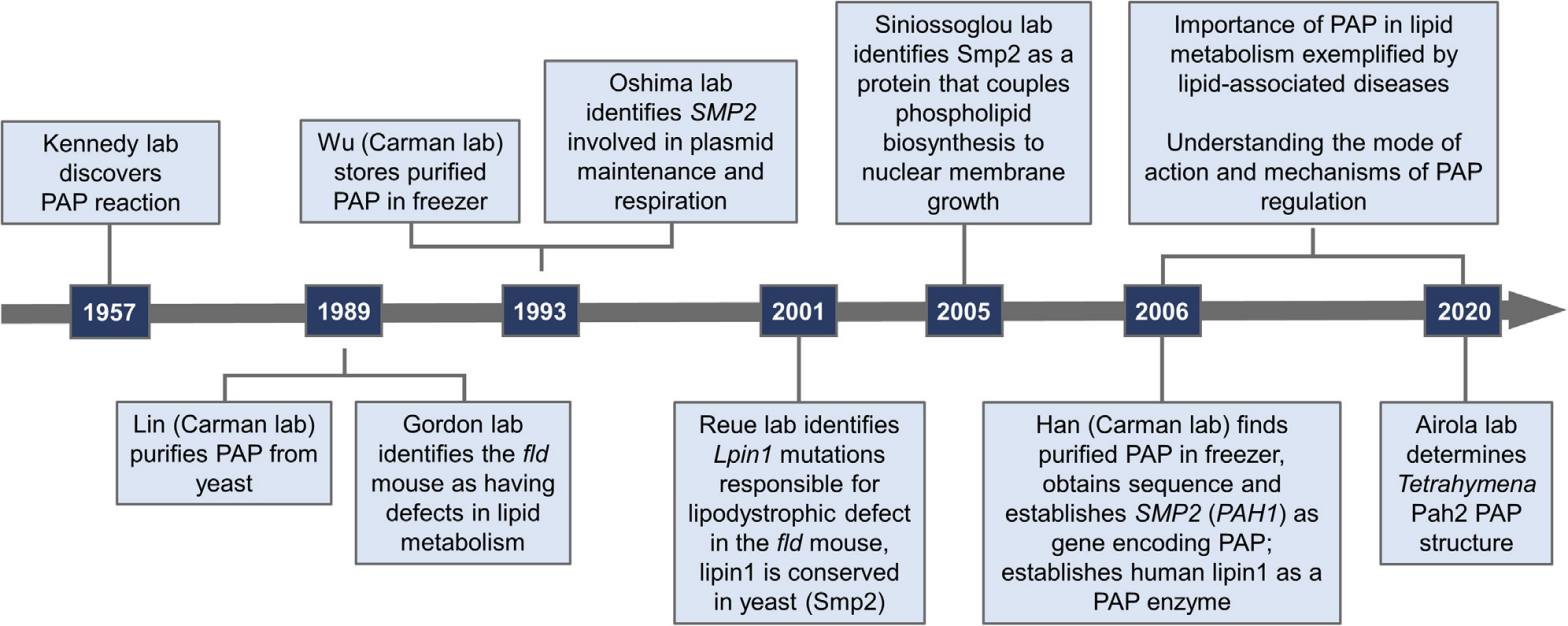
**PA phosphatase timeline.** The events depicted in the years 1957 (145), 1989 (69, 146), 1993 (90), 2001 (92), 2005 (100), 2006 (67), 2006 to 2020 (reviewed in (66, 91, 96), and 2020 (147)) are cited here. PA, phosphatidic acid; PAP, PA phosphatase.

Work from Karen Reue’s laboratory had already shown that Pah1 is a homolog of the mammalian protein lipin 1, which plays a role in lipid metabolism (92, 93). However, the molecular function of lipin 1 was unknown. Using human lipin 1 expressed in *E. coli*, we confirmed that the protein functions as a PA phosphatase (67, 94). Subsequently, the Reue laboratory, in collaboration with David Brindley, demonstrated that spliced variant forms of the mouse *Lpin1* gene, as well as the mouse *Lpin2* and *Lpin3* genes, encode PA phosphatase (95). Absence or mutation of PA phosphatase in yeast, mouse, and human results in a plethora of detrimental phenotypes and disease conditions; thus, the identification of the PA phosphatase genes in these and other organisms fueled an explosion of research on the enzyme (96, 97). Since its discovery, we have gone on to show that the expression of Pah1 PA phosphatase is regulated by nutrients and growth phase and that the enzyme is extensively phosphorylated for the regulation of its catalytic activity, subcellular localization, and protein stability (66, 97, 98) (Fig. 2). The timeline of PA phosphatase research is depicted in Figure 3. What has become clear is that too little or too much PA phosphatase activity is detrimental to cell physiology, and thus, the current work funded by the MIRA award (GM136128) is focused on understanding the mode of action and regulation of activity of Pah1 with the goal of identifying effector molecules that “fine-tune” the enzyme function in lipid metabolism. Additional key enzymes that work in conjunction with PA phosphatase include the Nem1–Spo7 complex (99) that dephosphorylates Pah1 (100, 101, 102) and the Dgk1 DAG kinase (103, 104) that phosphorylates DAG back to PA (Fig. 2). Studies on the regulation of these enzymes are also a focus of our current work.

## The mentors, mentees, and collaborators that helped shape my career

### Mentors

**Figure 4:**
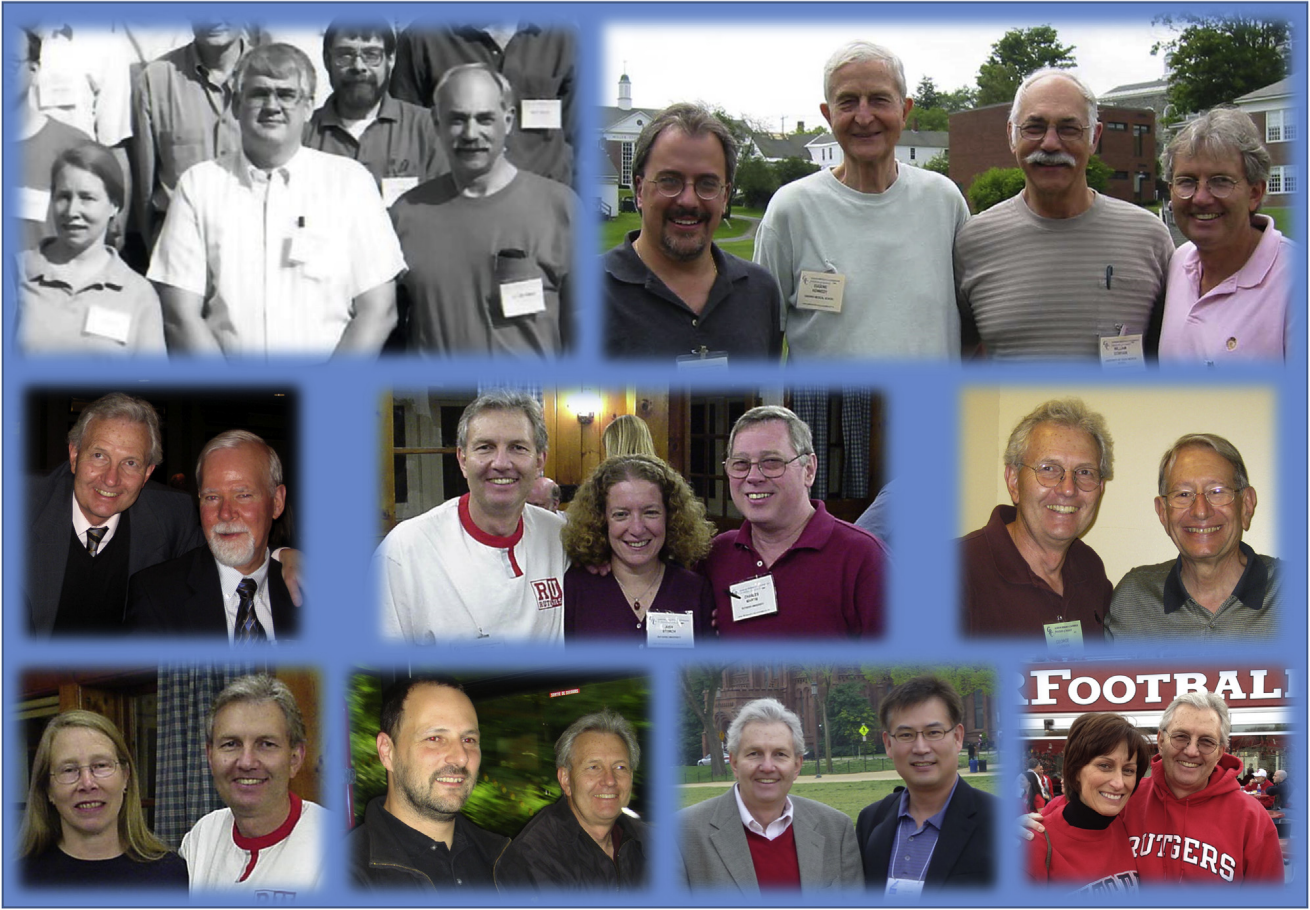
**Mentors and collaborators.***Top row*, *left*, Susan Henry, Bob Bell, and Bill Dowhan; *right*, Tony Fischl, Eugene Kennedy, Bill Dowhan, and me. *Middle row*, *left*, me and Dennis Voelker; *middle*, me, Judy Storch, and Chuck Martin; and *right*, me and Ed Dennis. *Bottom row*, *left*, Susan Henry and me; *middle left*, Symeon Siniossoglou and me; *middle right*, me and Gil-Soo Han, and *right*, Maureen Carman and me.

My mentors have supported my career through scientific advising and by recommending me to write review articles, speak at national and international meetings, organize scientific conferences and nominating me for appointments to editorial boards and for scientific awards. Bob Bell, Ed Dennis, Bill Dowhan, and Susan Henry have had the greatest influence on my career (Fig. 4). Bill’s mentorship did not end when I left his laboratory in 1978. In the early years, I probably called him on a weekly basis. His advice on research and grantsmanship continues to this day. While I had no formal relationships with Susan, Bob, or Ed, they always treated me like one of their own mentees in supporting my career. The early collaboration with Susan morphed into a lifelong friendship. Over the years, Susan has mentored me on approaches in yeast genetics and grantsmanship and always shared her strains and plasmids. While she was a professor at Albert Einstein College of Medicine, we often held joint laboratory meetings. When she moved to Carnegie Mellon University and then to Cornell University, our laboratories continued to interact on a regular basis. Ed, who is well known for his seminal studies on phospholipase A_2_ enzymes, lipid signaling, and lipidomics, is much involved with editorial functions and the organization of scientific meetings. He invited me to be a *Journal of Lipid Research* (JLR) Associate Editor (AE) and got me involved as an organizer of American Society for Biochemistry and Molecular Biology (ASBMB) annual meetings and conferences run by the Federation of American Societies for Experimental Biology (FASEB) and Keystone organizations. Bob is an icon in the field of lipid metabolism and signaling. During a poster session at the 1979 ASBMB annual meeting, Bob gave me constructive advice on how to prepare a competitive grant application, as my own early applications lacked focus or were overly ambitious. In fact, it was his advice to focus on the yeast PI synthase that resulted in my first NIH grant GM028140 awarded in 1980. Bob’s advice and encouragement, available by phone during his days at Duke University Medical School, were critical to our development of radioactive assays for PA phosphatase activity (105, 106) and the reconstitution of phospholipid synthesis enzymes into liposomes (107, 108). Moreover, as a JBC AE, he frequently asked me to serve as an ad hoc reviewer and ultimately recommended my appointment to the editorial board. I can never repay my mentors for supporting my career, except to “pay it forward” to my own mentees whether or not they receive formal training under my tutelage.

### Mentees

**Figure 5:**
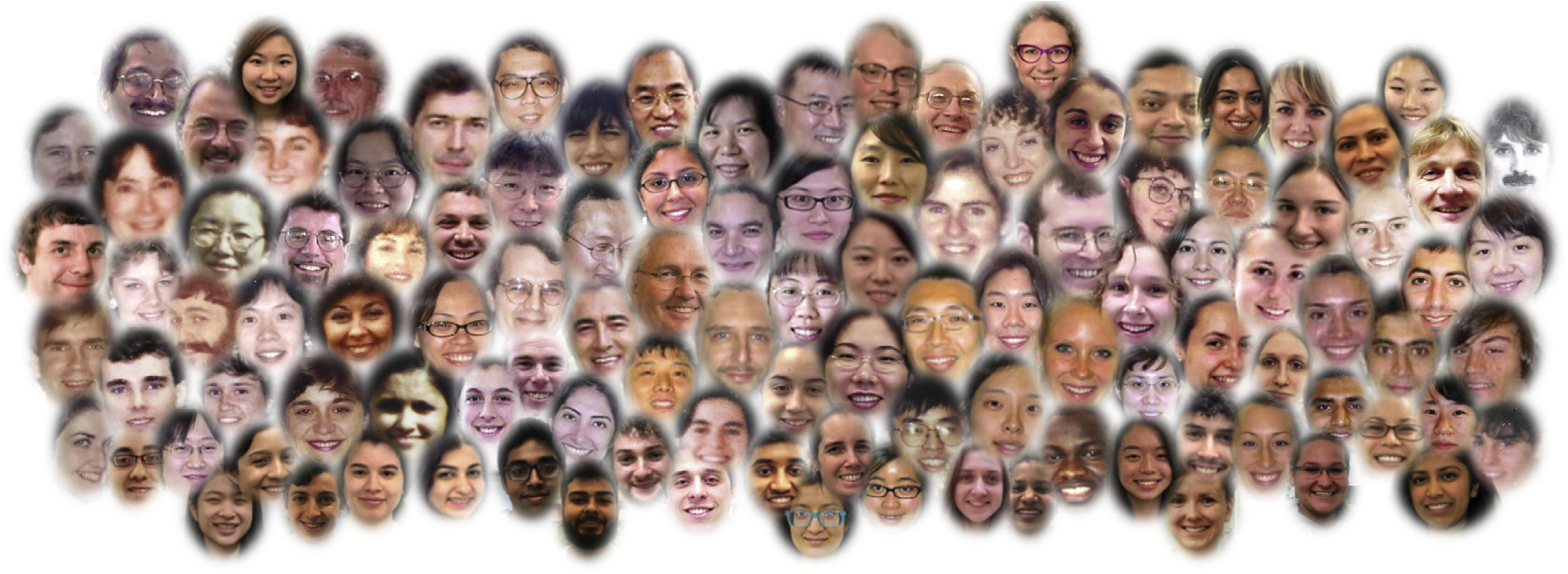
**Carman laboratory mentees**.

During my tenure at Rutgers University, I have had the honor of mentoring 30 postdoctoral associates, 52 graduate students, and 65 undergraduate research students. I do not have the space to tell a story about each one, but they are all very special to me (Fig. 5). I will mention, however, the mentees that particularly moved our research in new directions. To begin, there is my first graduate student Tony Fischl (Fig. 4). His work on the purification of PI synthase (42) and its reconstitution into liposomes (107) laid the groundwork for our studies on the PS synthase (44, 108) and CDP–DAG synthase (45). Mike Homann’s contributions led to the initial purification and characterization of PA phosphatase by Yi-Ping Lin. Wen-I Wu developed novel approaches to purify PA phosphatase, which ultimately led to the identification of the DAG pyrophosphate phosphatase (74), cloning of its structural gene *DPP1* (80), and studies on zinc-mediated regulation of *DPP1* (82) and other genes of phospholipid metabolism (83, 84, 85, 86, 87, 88, 89). Joe Stukey’s efforts led to the identification of a novel three-domain phosphatase motif (78) that facilitated the identification of the *DPP1* (80) and *LPP1* (109) genes that encode PA phosphatase enzymes, as well as the *LCB3* and *YSR3* genes encoding sphingoid base phosphate phosphatase enzymes discovered by other groups (110, 111, 112). Joe Nickels, who was talented with enzyme purification (113), brought photoaffinity labeling to the laboratory for mechanistic studies of PI 4-kinases (114). Our long-standing work on the phosphorylation of phospholipid synthesis enzymes was initiated by Tony Kinney through his work on PS synthase (115, 116). Weng-Lang Yang initiated our work on CTP synthetase by purifying the enzyme (117) and then performing work on its nucleotide- and phosphorylation-dependent regulation (118, 119, 120, 121). Finally, there is Gil-Soo Han who is arguably the most talented technical wizard I have ever known. His ingenuity led to the discoveries of the molecular functions of the Pah1 and lipin 1 proteins as PA phosphatases (67, 94), the *DGK1* gene encoding the novel CTP-dependent DAG kinase (103, 104), and the *LSB6* gene encoding PI 4-kinase (122). His insights into the biochemistry and molecular biology of lipid metabolism, and contributions to the research of other mentees in the laboratory, have been invaluable to our progress in recent years.

### Collaborators

I have been fortunate to have the opportunity to collaborate with many investigators. There are too many to acknowledge here, but I will mention Susan Henry, Dennis Voelker, and Symeon Siniossoglou (Fig. 4), with whom we published numerous papers. Susan Henry and I have collaborated in many studies on the transcriptional regulation of phospholipid synthesis (43, 49, 50, 51, 52, 53, 54, 55, 56, 57, 58, 59, 60, 61, 62, 63). I have learned much from her amazing insight into how phospholipid synthesis is regulated. A case in point was her 1998 hypothesis that PA is the key lipid metabolite that triggers the transcriptional regulation of lipid metabolism *via* the Opi1/Ino2-Ino4 (Henry) regulatory circuit (62, 64). It is gratifying that our biochemical and genetic studies on the Pah1 PA phosphatase and Dgk1 DAG kinase have confirmed this concept (66). Dennis Voelker, who I first met at the 1981 Gordon Research Conference on Lipid Metabolism, has been a terrific collaborator and friend. He is known for seminal studies on lipid trafficking and innate immunity. His laboratory helped us elucidate several gene–enzyme relationships in lipid metabolism by heterologous expression of the yeast genes in Sf9 insect cells (80, 109, 123, 124, 125). Finally, I acknowledge Symeon Siniossoglou, who first showed that Pah1 was involved in the regulation of phospholipid synthesis and nuclear/endoplasmic reticulum membrane growth (100). Once my laboratory defined the molecular function of Pah1 as PA phosphatase, we have collaborated extensively on studies of Pah1 and Dgk1 (101, 103, 104, 126, 127, 128, 129, 130, 131, 132, 133, 134, 135). One could not hope for a better collaborator than Symeon.

## Service to editorial boards, NIH study sections, and national/international meetings

Service to editorial boards, grant review panels, and the organization of scientific meetings is important to the advancement of science at large. As you will read below, it has been a major activity of my scientific career. Being a good scientific “citizen” has afforded numerous benefits that include career advancement, development of scientific relationships and collaborations, and opportunities to mentor early career scientists. These service activities, along with scientific accomplishments, have been recognized by the ASBMB by induction as an inaugural fellow of the society.

### Editorial boards

During the first part of my career, I received a great deal of reviewing experience by serving on the editorial boards of the *Journal of Food Biochemistry*, *Journal of Food Science*, *Applied and Environmental Microbiology*, and *Journal of Bacteriology*. I had always aspired to become an editorial board member of the JBC, the principal venue for seminal studies on the biochemistry and molecular biology of lipid-dependent enzymes and related processes. Under the leadership of Herbert Tabor, long-time EIC of the JBC, those appointed to the editorial board had to have published extensively in the JBC and served as an ad hoc reviewer for the journal. AE Bob Bell often called upon me to review papers during the late 1980s, and after “paying my dues” for several years, I was appointed (by recommendation of Bob Bell) to the board for a 5-year term in 1992 and then again in 1998. During these appointments, I worked closely with Bob Bell, and also other AEs, namely Alan Goodridge, Steve Prescott, Claudia Kent, David Russell (136)†, and Bill Smith (137)†, all icons in the field of lipid metabolism. I began my career at the level of AE/executive editor (EE) in 1994 when I was appointed (by the recommendation of Claudia Kent) EE of *Analytical Biochemistry*. I still serve in this role where I primarily handle papers on methods in lipidology. In 2003, and again in 2017, I was appointed (by the recommendation of Ed Dennis) AE of the JLR, and in 2004, EE (by the recommendation of Dennis Vance (138)†) of *Biochimica et Biophysica Acta* (BBA)-*Molecular and Cellular Biology of Lipids*. Juggling editorial responsibilities for three journals prepared me, well sort of, for my appointment (by the recommendations of David Russell and Bill Smith) as an AE of the JBC in 2006. The work load for the JBC was enormous; Herb assigned me 30 to 40 papers/month, and consequently, I had to resign from the AE and EE boards, respectively, of the JLR and BBA. At first, I was intimidated by the stature of other members of the JBC AE board. They were all well-known leaders in their respective fields. My insecurities were alleviated by Dick Hanson, Bob Simoni, Herb Tabor, and Martha Vaughan who welcomed me to the AE board and provided guidance when needed. Of course, Bill Smith and David Russell were very supportive. During my tenure (2006–2019) as a JBC AE, I followed Herb’s guiding principle of giving authors the chance to improve their manuscript and thus be made worthy of publication in the JBC. In the last few years of my tenure, I chaired the “Reflections and Classics” committee and worked closely with Herb, who held emeritus EIC status with the journal.

A tremendous benefit of my editorial board service has been the friendships developed with other AEs/EEs. These include Howard Goldfine (139)† and Carlos Hirschberg at *Analytical Biochemistry*, Al Merrill at JLR, Fritz Spener at the BBA, and David Russell, Bill Smith, and Dennis Voelker at the JBC. Perhaps the most important relationships developed were those with members of the editorial boards of these journals, those who I relied on to aid me in difficult editorial decisions. Moreover, being an AE/EE also allowed me to recommend early career scientists to editorial boards, and so, I had the opportunity to mentor them as I had been aided by my mentors.

### NIH study sections

My first experience in reviewing grant applications came in 1984 when I served (at the recommendations of Bob Bell and Bill Dowhan) as an ad hoc member on the Physiological Chemistry (PC) Study Section of the NIH. This study section handled a broad range of topics including the biochemistry and molecular biology of proteins, carbohydrates, and lipids. I recall that upon return from the Yeast Genetics and Molecular Biology meeting in Edinburgh finding a box with about 130 grant applications. In those days, there was a 25-page limit for the experimental plan and no limit on the amount of materials in the supplement and updates, and the updates kept coming up until the day of the meeting. I was assigned 13 applications as either the primary or secondary reviewer. One evening when reading the applications at my dining room table, I came to tears because of the amount of work involved in preparing for the meeting. The PC Study Section roster included an amazing cast of famous scientists; I was afraid to speak in fear that I would be off target in evaluating my assigned applications. I guess I did a good job because I was invited back for ad hoc service in 1986 and 1987, and then appointed (as recommended by Gerald Carlson) as a regular member in 1988. I agreed to serve a second term in 1998.

A major lesson learned from serving on the PC Study Section was to support early career investigators and abide by the so-called “Harland Wood Factor.” Harland Wood (1907–1991) was an internationally known scientist from Case Western Reserve University and National Medal of Science recipient who served on the President's Science Advisory Committee under Lyndon B. Johnson and Richard Nixon. Wood felt that it was the duty of PC Study Section members to apply common sense and wisdom to ensure that new investigators could get grants to move their careers along. (The application of my first NIH grant GM028140 was reviewed by the PC Study Section in 1980; I’m sure it received a boost thanks to the Harland Wood Factor).

In 2003, I served on the NIH Study Section Boundaries Team for Biological Chemistry and Macromolecular Biophysics Integrated Review Groups. This meeting led to the disbandment of the PC Study Section and creation of the Biochemistry and Biophysics of Membranes Study Section, of which I later became a regular member (2011–2015). During the intervening years, I served on several NIH special emphasis panels. The enormity of time and work notwithstanding, the major benefit of serving on the study sections was the interaction with a cadre of incredibly talented scientists (*e.g.*, Gerald Carlson, Wonhwa Cho, Joseph Eichberg, Richard Gross, Suzy Jackowski, Lauri Kaguni, Anne Kenworthy, Claudia Kent, Richard Kolodner, Bill Merrick, Andrew Morris, Alexandra Newton, Peter Roach, David Russell, Suzanne Scarlata, and Lukas Tamm). Some of the interactions led to fruitful collaborations and those I met who were not associated with the lipid field were particularly helpful when I was called upon to chair and organize the ASBMB annual meetings in 2001 and 2006 (see below). The Scientific Review Officer/Executive Secretary of an NIH study section is generally not recognized for the work that goes into organizing the rosters and meetings, and preparing the summary statements (“pink sheets”) for the applications. Stanley Burrous and Richard Panniers of the PC Study Section, Nuria Assa-Munt of the Biochemistry and Biophysics of Membranes Study Section, and Don Schneider of special emphasis panels were outstanding administrators that deserve special mention because they always had the best interests of the applicants in mind during the meetings. When thinking about NIH administrators, I must also acknowledge Jean Chin who was the supportive Program Official of my NIH grant GM028140.

### National/international meetings

Another important service activity that has played a major role in my career is the organization of scientific meetings. The first major meeting that I organized was the 1993 Gordon Research Conference on Lipid Metabolism (now called Molecular and Cellular Biology of Lipids). This was one of the greatest honors in my career because the nomination for election is made by the past chairs of the conference. My nominators included Bob Bell, Ed Dennis, Howard Goldfine, Claudia Kent, Chris Raetz, and David Silver. Organizing and raising funds for the meeting was an invaluable experience. Among the speakers were Bob Bell, Lew Cantley, Ed Dennis, Bill Dowhan, Eugene Kennedy, Richard Gross, Yusuf Hannun, Susan Henry, Claudia Kent, Bob Lester, Phil Majerus, Al Merrill, Dick Pagano, Chris Raetz, Sue Goo Rhee (140)†, David Russell, Dennis Vance, Dennis Voelker, and Karl Wirtz. What a thrill it was for so many icons in the field to accept my invitations to speak. The Gordon Research Conference on Lipid Metabolism is my favorite meeting, and I have not missed one since 1981. Additional small meetings that I have organized or coorganized include the Keystone Symposium “Cell activation and signal transduction: lipid second messengers IV” (2000), ASBMB Satellite/Theme Meetings “Molecular characterization of membrane lipid metabolism” (1998), “Membrane lipids and cell function” (2001), and “Biochemistry and molecular biology of lipids” (2006), and the FASEB Science Research Conference “Phospholipid cell signaling and metabolism in inflammation and cancer” (2014). The organization of the small meetings prepared me for the huge job of organizing the scientific programs of the ASBMB annual meeting in 2001 and the Centennial meeting in 2006. I had terrific co-chairs Alexandra Newton and Lauri Kaguni for these meetings. Together, we recruited outstanding scientists to organize the various themes/sessions of the annual meeting. We also had a great deal of help from the annual meeting program planning committees and the staff of the society. Barbara Gordon (Executive Director) and Joan Geiling (Meetings Manager) were particularly instrumental to the success of these meetings. Additionally, I have served as chair of the ASBMB Meetings committee (2002–2004), the FASEB Science Research Conference Advisory Committee (2003–2012), and the steering committees of the International Conference on the Bioscience of Lipids (2010–2013, 2018–2021) and the international Yeast Lipid Conference (2005–2021). Like other service activities, a major benefit of organizing meetings has been the scientific relationships developed, and importantly, the ability to advance the careers of early career scientists by inviting them as speakers at the meetings.

## Rutgers Center for Lipid Research

I have had supportive colleagues in the Department of Food Science at Rutgers, but their research areas were far from mine, and I longed for colleagues with similar interests in lipids. Although Chuck Martin (hired by biology in 1978) and Judy Storch (hired by nutrition in 1992, and who I first met at the 1985 Gordon Research Conference on Lipid Metabolism) had interests in lipids, we seldom saw each other on campus except for when we served together on dissertation committees. Instead, the three of us would meet regularly at the Gordon Research Conference or at annual ASBMB meetings. Over the years, more investigators who had interests in lipids joined Rutgers; however, they were from various departments across the campuses in New Brunswick, Piscataway, Newark, and Camden. In the summer of 2007, Chuck, Judy, and I brainstormed about forming a center that would bring Rutgers lipid researchers together. Bob Goodman, then dean of the School of Biological and Environmental Sciences, agreed to let me organize the Rutgers Center for Lipid Research (RCLR), which is now part of the New Jersey Institute for Food, Nutrition, and Health. Charter members of the center included Dawn Brasaemle, Joe Dixon, Ariel Igal, Chuck Martin, Rich Mendelsohn, Judy Storch, Phil Yeagle, and myself. Since its inception in 2008, the RCLR has grown to include 39 members (∼140 including students and postdocs) spread across the University and Medical School located on different campuses. The RCLR promotes multidisciplinary research on the biochemical, biophysical, cellular, and molecular mechanisms of lipid metabolism, and extension of these endeavors to elucidate the underpinnings of lipid-related diseases such as obesity, lipodystrophy, diabetes, and heart disease. Most importantly, the center fosters interaction among faculty, postdoctoral associates, and students by holding monthly research meetings where postdoctoral associates and students present their research and receive constructive feedback in a warm and friendly atmosphere. Moreover, we provide small grants and travel support to students and postdoctoral associates. We hold an annual symposium and a monthly seminar series that bring renowned scientists to Rutgers. I’m also proud that in 2016, the RCLR founded the Big Ten Academic Alliance Lipid Symposium; this meeting brings lipid researchers at Big Ten schools to interact on a regular basis. One of the companies that supports RCLR and Big Ten symposia is Avanti Polar Lipids; Walter Shaw, who is the founder and past owner of the company, has been supportive of lipid meetings held throughout the world and deserves special mention.

## Carman prize in lipids

I am very fortunate that my research accomplishments have been recognized by receipt of several awards, which include the ASBMB-Avanti Award in Lipids and the American Oil Chemists' Society–Supelco/Nicholas Pelick Research Award. With proceeds from these awards, along with funds from various Honoria and donations from past students, the George M. and Maureen D. Carman Prize in Lipids was endowed by the Rutgers University Foundation. The prize, which is awarded for research achievement in lipid biochemistry, provides financial assistance to graduate students and postdoctoral associates.

## Carman laboratory mission statement and mottos

Stephen R. Convey’s book “The 7 Habits of Highly Effective People” (141) has been a terrific resource of approaches to solve professional (and personal) challenges. One of Convey’s suggestions is to develop a detailed mission statement, and that is what we did. The mission statement reflects Carman laboratory core values of honesty and integrity, and willingness to share with others; it is prominently placed on the wall of the laboratory conference room and the Carman web page. Our core values are reflected in these mottos: (1) don’t assume anything; (2) just because it is published doesn’t mean it is fact; (3) you don’t detract from your own accomplishments by acknowledging those of others; and (4) pay it forward.

## Conflict of interest

The author declares that he has no conflicts of interest with the contents of this article.

## Footnotes

† Reflections article.
